# An Update on the Ophthalmologic Features in the Phakomatoses

**DOI:** 10.1155/2016/3043026

**Published:** 2016-07-17

**Authors:** Solmaz Abdolrahimzadeh, Andrea Maria Plateroti, Santi Maria Recupero, Alessandro Lambiase

**Affiliations:** ^1^Ophthalmology Unit, DAI Testa/Collo, Azienda Policlinico Umberto I, Department of Sense Organs, University of Rome “Sapienza”, Viale del Policlinico 155, 00161 Rome, Italy; ^2^Ophthalmology Unit, St. Andrea Hospital, NESMOS Department, University of Rome “Sapienza”, Via di Grottarossa 1035-1039, 00189 Rome, Italy

## Abstract

Neurofibromatosis type 1, tuberous sclerosis complex, and Von Hippel-Lindau disease, historically classified as the phakomatoses, are hereditary multisystem disorders characterized by the presence of hamartoma, which carry the risk of malignant transformation. The alteration of tumor suppressor genes seems to be at the basis of their pathophysiogenetic mechanism. Lisch and choroidal nodules in neurofibromatosis type 1, retinal astrocytomas in tuberous sclerosis complex, and retinal capillary hemangioma in Von Hippel-Lindau disease are the principal ophthalmic hamartomatous manifestations. The advent of novel imaging techniques such as near infrared reflectance and optical coherence tomography has provided unprecedented insight on the choroidal and retinal features of these diseases. These methods have improved early diagnosis and the ongoing surveillance in these conditions. Among an array of treatment modalities, antivascular endothelial growth factor therapy has been used in the management of retinal hamartomas but results have been varied. This review is an update on the pathophysiogenetic mechanisms, ophthalmic manifestations, and novel treatment strategies in the phakomatoses with emphasis on the role of imaging techniques.

## 1. Introduction

Van der Hoeve in 1932 included neurofibromatosis type 1 (NF1), tuberous sclerosis complex (TSC), and Von Hippel-Lindau disease (VHL) in a group named “phakomatoses.” The conditions were defined as hereditary multisystem disorders, which share the common characteristics of the presence of “spots, tumefactions, and cysts” which were described as “phakomata” (from the Greek term “phakos” for mother-spot), which proliferate and carry the risk of malignant transformation [1]. Over the years there has been much debate and some authors have suggested the inclusion of other ophthalmic diseases with neurocutaneous manifestations in the group of phakomatoses [2–6], but advances in molecular genetics provide evidence of the role of tumor suppressor genes in the pathophysiogenetic mechanisms of NF1, TSC, and VHL disease, which all have a familial pattern of inheritance, variable expressivity, and multisystem tumors with a risk of malignant transformation and can be considered the “true phakomatoses” [7–9].

Recent progress in multimodal imaging techniques, like near infrared reflectance (NIR) and optical coherence tomography (OCT), has enhanced our knowledge on the retinal manifestations of these diseases, providing unprecedented information on the morphological aspects of hamartomas. These methods have improved early diagnosis and the ongoing surveillance in these conditions. Indeed, facilitated and noninvasive imaging with NIR has led to propose choroidal nodules as an additional diagnostic criterion in NF1, which aids diagnosis in children and in uncertain cases [10]. Peripapillary retinal nerve fiber layer imaging with OCT is potentially a rapid tool for monitoring children with NF1 as regarding optic pathway glioma [11]. Furthermore, OCT has been used to better classify retinal alterations in TSC [12]. Novel therapeutic strategies involve the tentative use of antivascular endothelial growth factors (anti-VEGF) in the management of hamartomas and/or the complications arising from their presence.

The present paper is a detailed review of the ophthalmological features in the phakomatoses with particular attention to novel imaging findings and ongoing management methods.

## 2. Neurofibromatosis Type I

NF1 is an autosomal dominant disease due to deletions or mutations of the neurofibromin gene located on chromosome 17p11.2 [13]. The prevalence of the disease is about 1 : 3000 and is not based on gender, race, ethnic group, and geographical area [14]. NF1 has complete penetrance and variable expressivity but in about 50% of individuals the condition is due to de novo (spontaneous) mutations [15–19].

Ophthalmological features included in the NIH diagnostic criteria of NF1 are Lisch nodules and optic pathway gliomas (OPG) [16]. Other ocular structures involved include the orbit, eyelid, conjunctiva, cornea, anterior chamber, ciliary body, and retina. In patients with absence of the greater wing of the sphenoid bone, the most dramatic clinical sign is a pulsating proptosis, which is synchronous with heartbeat. Other signs linked to dysplasia can be microphthalmus and enophthalmos [20]. Upper eyelid plexiform neurofibromas are unilateral and are observed in the first two years of life. They can increase in dimensions leading to asymmetrical ptosis with the typical freeboard “italic S” course of the eyelid margin. In some cases, enlargement can lead to elephantiasis and extend to the temporal and frontal areas creating facial asymmetry. Although various surgical approaches have been attempted, results are disappointing because of copious intraoperative bleeding and tendency to relapse [6, 20–22]. Sporadic conjunctival neurofibromas and typical asymptomatic hypertrophia of intrastromal corneal nerves (observed in one out of four patients) have been reported [6]. Corneal nerve thickening can possibly be due to the intrinsic genetic alteration of the disease with axon and Schwann cell abundance [23]. Similar to other genetic diseases with corneal nerve thickening, epithelial changes may result from possible corneal nerve dysfunction and should be evaluated when considering refractive corneal surgery in these patients [24, 25].

**Figure 1:**
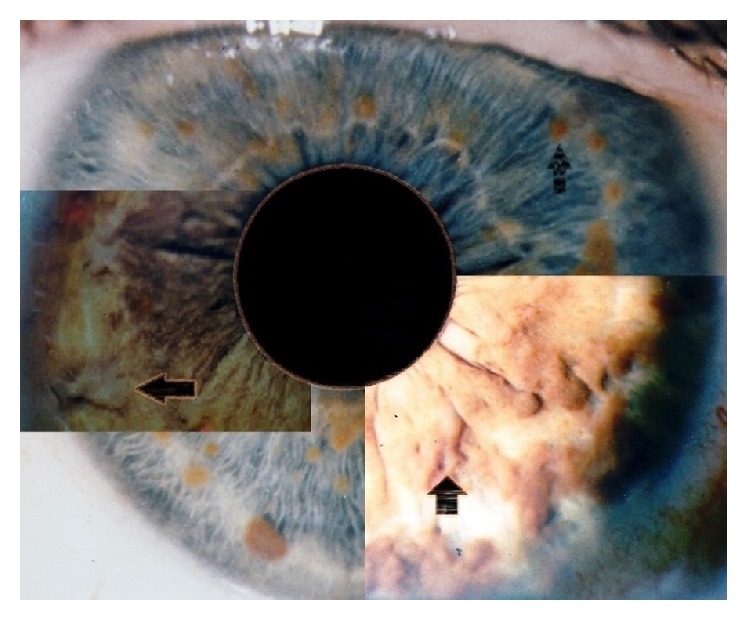
Morphological presentations of Lisch nodules. The upper and background image shows some shaped choroidal nodules. The image on the left shows nodules with ragged borders. The image on the right shows nodules with confluency (black arrows indicate Lisch nodules).

Lisch nodules of the iris represent the main ocular manifestation of NF1. They usually appear from the age of 2 years onwards and approximately 50% of children present nodules; by adulthood this number increases to over 90% [26–29]. The number of Lisch nodules is correlated with choroidal nodules [30] and we found that Lisch nodules are positively correlated with cutaneous neurofibromas with increasing age [27]. Lisch nodules are often pigmented and their color may vary from creamy white in dark irides to brown in blue and green irides. We observed that they can vary from dome-shaped nodules to masses with ragged borders and various grades of confluency (Figure 1) [27].

**Figure 2:**
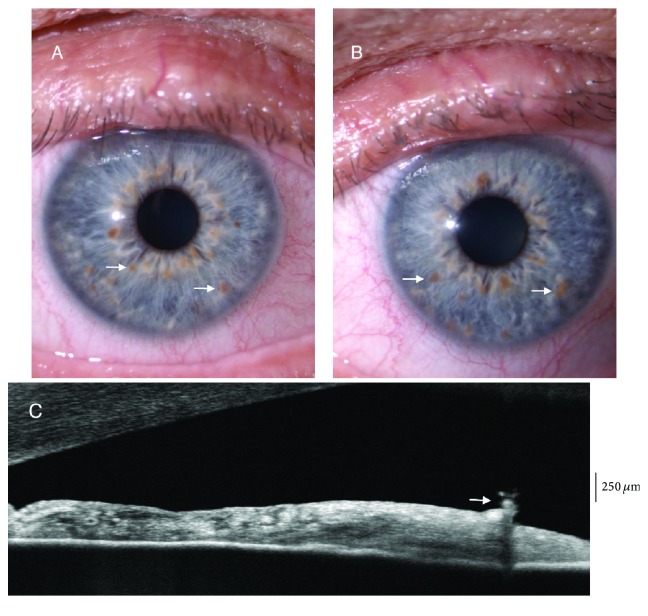
Lisch nodules in neurofibromatosis type 1. Slit lamp (A, B) and anterior segment optical coherence tomography images (C), arrows indicate Lisch nodules.

Ragge et al. reported that they were more frequently localized in the inferior hemifield due to the sunlight-shielding effects of the upper eyelid [28]; however, in our study we did not find a predilection for any specific area [27]. Lisch nodules are easily observed with slit lamp examination; however, they can be difficult to detect due to poor cooperation of young patients, when they are small or flat, or when they are localized in the iris crypts [31] (Figure 2). Lisch nodules should not be confused with iris mammillations found in ocular melanosis which are smooth and homogenously distributed iris nodules that are commonly unilateral [32, 33].

Glaucoma onset is rarely seen in NF1, although multiple pathogenic mechanisms related to angle closure have been proposed. The most commonly reported mechanisms are infiltration of the anterior chamber by neurofibromas that obstruct the angle, secondary angle closure by neurofibromatous cysts or increased thickness of the ciliary body and choroid, neovascular glaucoma, and developmental angle abnormalities [34–36]. Optical coherence tomography of the anterior chamber and ultrasound biomicroscopy can show Lisch nodules and abnormalities of the ciliary body and chamber angle involved in glaucoma onset [37–39]. Morales et al. found ipsilateral glaucoma and increased axial length in 23% of 95 patients with orbitofacial involvement and associated findings were pigmentary patches or anterior synechia and thickening of the ciliary body [40]. Therefore, tonometry and visual field analysis in patients with NF1 is advisable although this is not always easy to perform in younger patients [41–44].

**Figure 3:**
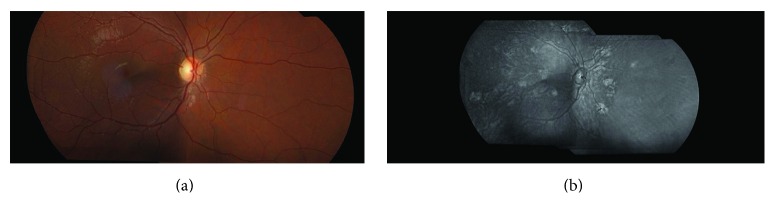
Color fundus photograph (a) and near infrared reflectance fundus image (b) in a patient with NF1. Patchy and rounded choroidal alterations are most apparent on infrared reflectance images.

Choroidal abnormalities of NF1 are often difficult to detect via fundus biomicroscopic examination and fluorescein angiography (Figure 3).

**Figure 4:**
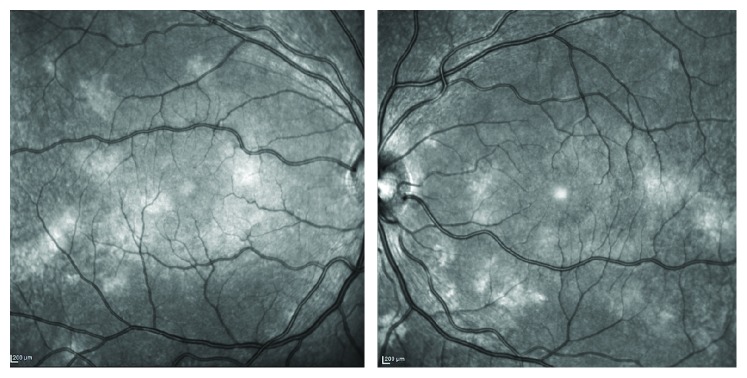
Near infrared reflectance showing choroidal alterations in neurofibromatosis type 1. The typical rounded or patchy alterations are clearly shown.

Indocyanine green angiography in NF1 patients shows delayed perfusion of the choriocapillaris in areas corresponding to choroidal nodules [45, 46]. Yasunari et al. used infrared monochromatic light to show multiple bright patchy choroidal alterations corresponding to choroidal nodules of the posterior pole in 33 eyes of 17 patients with NF1 [46]. Recently, Viola et al. evaluated choroidal alterations in NF1 patients using near infrared reflectance (NIR) in 190 eyes and found nodules in 82% of patients. They established a cut-off value of 1.5 nodules to make a diagnosis and proposed to include choroidal nodules among the diagnostic criteria [47] (Figure 4). NIR imaging with OCT to detect choroidal alterations has widened the armamentarium of ophthalmologists in the diagnostic process in NF1. OCT instrumentation is a valuable adjunct in the examination of children who cannot collaborate due to their young age and condition [10].

**Figure 5:**
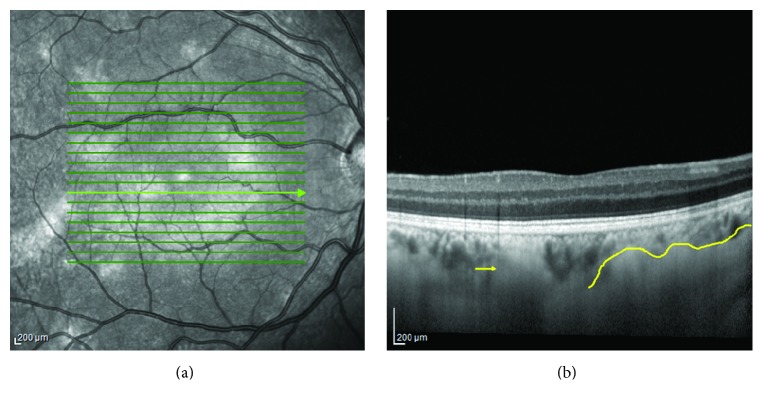
Raster (a) and corresponding cross-sectional (b) enhanced depth spectral domain optical coherence image of choroidal nodules in neurofibromatosis type 1. The delineated area shows a laterally extending placoid type choroidal nodule whereas the arrow indicates a dome-shaped nodule.

The enhanced depth imaging function of spectral domain OCT allows the visualization of deeper ocular structures than traditional OCT as the peak sensitivity is placed behind the retinal pigment epithelium [48]. Ayata et al. performed both longitudinal (B-scan) and transverse (C-scan) OCT imaging and observed choroidal nodules at different depths in the choroid and demonstrated that the overlying retina showed changes in thickness [49]. Rao and Choudhry found that the choroid over nodules had a loss of lucency suggesting vascular compression [50]. We previously reported on choroidal nodules in 38 eyes of 19 patients with NIR imaging and cross-sectional EDI-OCT images and showed that NIR detected choroidal alterations corresponded to two types of hyperreflective choroidal nodules on EDI-OCT. Well-delineated, bright, rounded shape alterations on NIR corresponded to “dome-shaped” hyperreflective formations on OCT-EDI and poorly defined patchy alterations corresponded to rather flat, irregularly shaped, hyperreflective “placoid” formations [26] (Figure 5).

**Figure 6:**
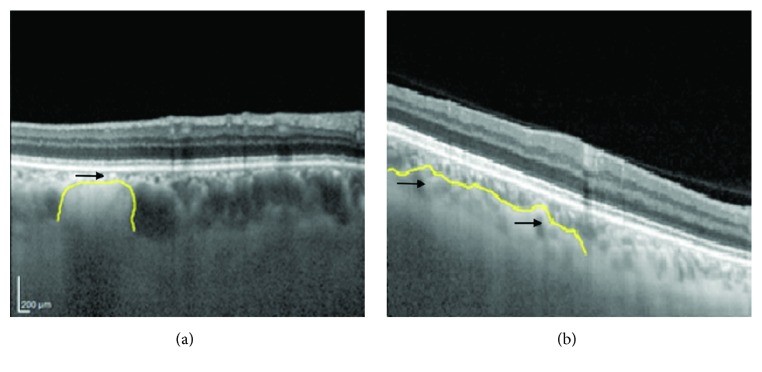
Optical coherence tomography-enhanced depth imaging of choroidal nodules and overlying choroidal vasculature features. (a) Delineated dome-shaped nodule; note the compressed appearance of choroidal vasculature and the absence of large caliber vessels. (b) Delineated placoid nodule; note the random localization of large caliber vessels among the irregular propagations of the placoid nodule (from [26]).

Moreover, through manual segmentation measurements of retinal layer and choroidal thickness using OCT software, we demonstrated generalized choroidal and retinal thinning in patients with NF1 [26]. We showed that choroidal vasculature above nodules was altered in NF1 (Figure 6) and as the choroidal vasculature is the main vascular support for the outer retina, we hypothesized that compromised choroidal blood flow can lead to an alteration of retinal trophism [26].

**Figure 7:**
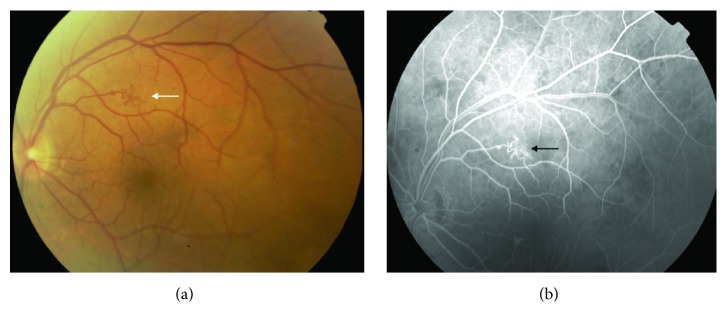
Retinal microvascular abnormality with “hemangioma-like” or “ball of thread” appearance in NF1. The retinal microvascular abnormality is shown with an arrow on the (a) fundus photograph and on the (b) fluorescein angiography image (modified from [52]).

Retinal microvascular anomalies consisting in small (second or third order venule) tributaries of the inferior or superior-temporal vein denominated “corkscrew retinal vessels,” “hemangioma-like,” or “ball of thread” retinal microvessels have been reported and these seem to be overlying areas corresponding to choroidal nodules as shown by NIR imaging (Figure 7) [51, 52].

Other retinal alterations such as multiple retinal capillary hemangiomas, astrocytic hamartomas, and, rarely, combined hamartomas of the retina and the retinal pigment epithelium (CHRRPE) were described [53]. OCT features of CHRRPE have been reported to include preretinal membranes, retinal striae, and a disorganization of the retinal layers including the retinal pigment epithelium and photoreceptors-ellipsoid zone [54, 55].

Optic pathway gliomas (OPG) have the most dramatic impact on visual acuity in NF1 patients. They are present in 15%–20% of children with NF1 before the age of eight [56]. These tumors are mainly grade I glial neoplasms, termed pilocytic astrocytomas, which are histologically similar to gliomas that arise sporadically in individuals without NF1 [56]. Although many OPGs are asymptomatic, one-third to one-half can cause clinical symptoms, usually resulting in reduced vision, proptosis, visual field alterations, color alterations, and precocious puberty [57]. King et al. studied 51 NF1 children with symptomatic OPG where 39% presented decreased visual acuity, 26% had proptosis, 20% had precocious puberty, and 12% presented strabismus. Only 10% of patients showed visual field alterations [58]. Visual field analysis is important in the screening of patients both for glaucoma and for possible OPG [59]. However, in young children this test is not always simple to carry out due to the young age of patients, which, however, improves with learning and increasing age [60, 61].

**Figure 8:**
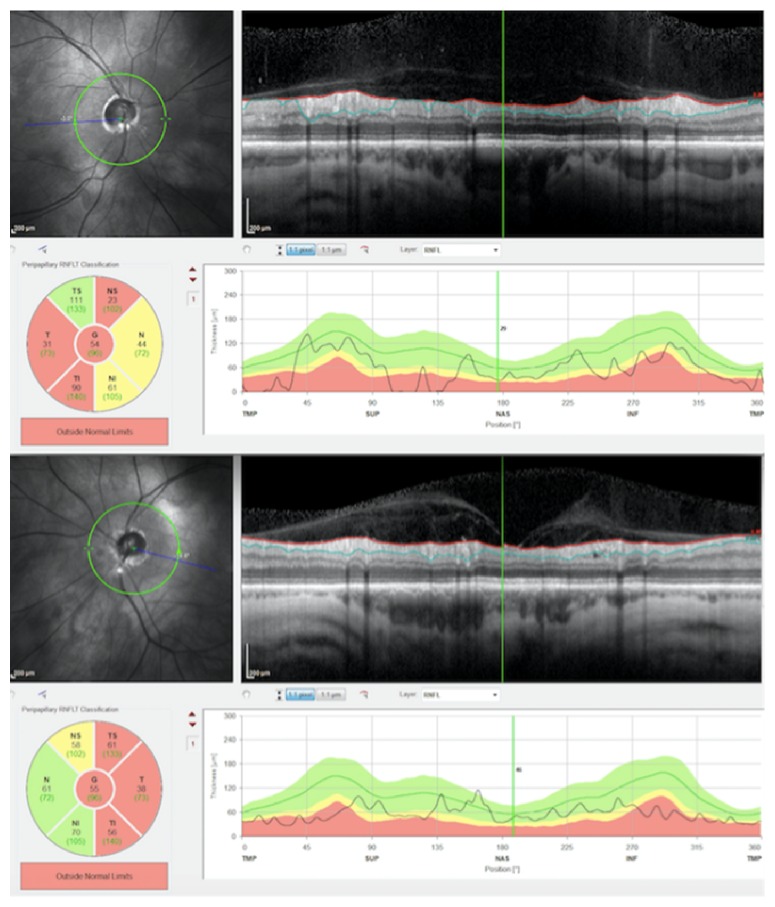
Spectral domain optical coherence tomography of the peripapillary retinal nerve fiber layer in an adult patient with neurofibromatosis type 1 showing reduction of thickness in both eyes.

Several studies demonstrated a correlation between OPGs in NF1 affected children and OCT evidenced thinning of the retinal nerve fiber layer (RNFL) [57, 62, 63]. Gu et al. found a reduction of thickness in the ganglion cell layer-inner plexiform layer (GCL-IPL) in children with NF1 and OPGs and suggested this as a more reliable measure of neuronal loss with respect to RNFL thickness [64]. Current guidelines for OPG management include magnetic resonance imaging (MRI) in patients presenting with signs of OPG [65]. Chang et al. proposed the use of OCT in children with OPG [63], although the OPG task force does not make a recommendation for routine OCT testing [65]. A variation in OPG size is not always well correlated with visual outcomes; therefore, a reduction in visual acuity or alteration of visual field implicates a need to initiate or alter treatment strategy. Results of visual acuity and visual field assessment in children are not always reliable due to their young age. Furthermore, children have difficulty in cooperating with standard table mounted OCT devices; thus Avery et al. reported on the use of handheld OCT in children with OPG under sedation [11]. These authors found highly reproducible thickness measurements of the GCL-IPL and suggested that a 10% change in the thickness of this layer should be deemed clinically significant [11]. In a recent investigation at the ophthalmology unit of the University of Rome, we used spectral domain OCT to evaluate macular neuronal and axonal thickness values in adult subjects with NF1 without OPG. We found that the peripapillary RNFL, macular RNFL, and GCL-IPL were reduced in thickness (Figure 8).

We speculated that this could be due to the age related, chronic nature of NF1 or that choroidal and retinal thinning could also involve the macular and peripapillary RNFL and the GCL-IPL [26, 66]. Furthermore, we found that the RNFL and GCL-IPL thickness values were correlated in NF1 patients; thus, we suggested the peripapillary RNFL/GCL-IPL index as a possible parameter in monitoring disease evolution [66].

Table 1 summarizes the clinical findings and diagnostic procedures in NF1.

High levels of VEGF have been demonstrated in plexiform neurofibromas and malignant peripheral nerve sheath tumors [67]. Interestingly since VEGF is produced by Schwannoma tumor cells, systemic bevacizumab therapy has been in the management of vestibular schwannomas in neurofibromatosis type 2 [68, 69] but further research is warranted to evaluate anti-VEGF therapy in NF1.

## 3. Tuberous Sclerosis Complex

Tuberous sclerosis complex is a multisystemic disease outlined by hamartomas involving the central nervous system, eye, skin, kidneys, heart, lung, and liver. This condition was first described by Bourneville in 1880 who documented typical tuber-like cerebral lesions in the human brain at postmortem examination [70]. In 1932, Van der Hoeve described the retinal changes [1]. The mutations for the disease, discovered in 1987, are* TSC1* and* TSC2* located on chromosomes 9q34 and 16p13, which encode for hamartin and tuberin, respectively [71]. The incidence of the disease is estimated to range from 1/6800 and 1/15000 and the prevalence is 1/10,000 with 50% to 84% of the cases being sporadic [71].

**Figure 9:**
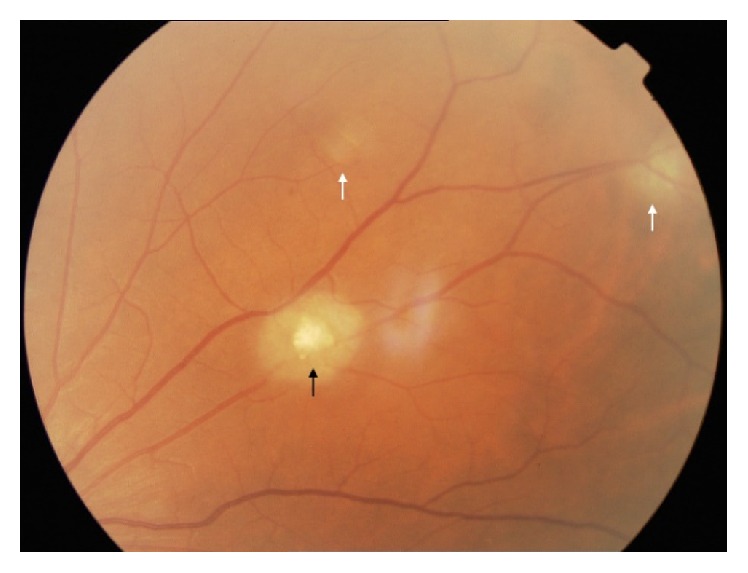
Astrocytic hamartoma of the retina in a patient with tuberous sclerosis complex. Multinodular or older tuberous body (black arrow). Translucent, blurry lesions or “younger tuberous bodies” in the periphery (white arrows).

**Figure 10:**
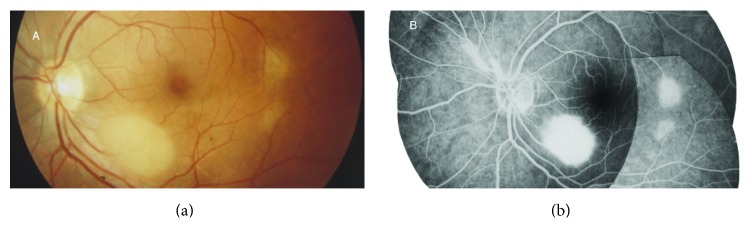
Astrocytic hamartoma of the retina in a patient with tuberous sclerosis complex. “Younger tuberous bodies” seen as smooth, flat translucent, or blurry lesions and “transitional tuberous bodies” seen as smooth, relatively flat, noncalcified, grey/white lesions with an oval or circular shape. (a) Fundus photograph and (b) corresponding fluorescein angiography image (from [6]).

The typical retinal lesions are astrocytic hamartomas, which can be the first manifestation of disease and are among the major criteria for diagnosis. They have been reported in 44% to 48% of patients in two large case series [72, 73]. Retinal hamartomas characterized by fibrotic astrocytes with small oval nucleus and long cytoplasmic extensions were reported by Rowley et al. in 44 of 100 (41%) patients, with bilateral presentation in 15 cases (34%). These hamartomas are divided into three basic morphological types: smooth, relatively flat, noncalcified, grey, translucent lesions with an oval or circular shape also known as “younger tuberous bodies”; elevated, multinodular, calcified, opaque lesions similar to mulberries also named “older tuberous bodies”; transitional lesions characterized by both features [72] (Figures 9 and 10).

The “younger tuberous bodies” are not easy to identify with ophthalmoscopy due to their translucent or blurry aspect. At times the only sign that can help to identify these lesions is a perilesional circular reflex. They are often localized near a vessel, which gives it an interrupted aspect. These vessels are usually frail and can lead to vitreous hemorrhage. The “older tuberous bodies” are usually peri- or epipapillary and can be confused with optic disc drusen. Their size can range between one-half and four optic disk diameters. Their structure has a high acoustic density at B-scan ultrasonography due to calcifications in the lesions. The transitional lesions have a smooth margin appearance and can be multinodular in the center. Fluorescein angiography can help to recognize the vascularization pattern and to distinguish younger from older lesions. In most cases visual acuity is not affected due to extramacular localization of lesions. Visual field defects and a progressive loss of visual acuity can be associated with optic disc hamartomas for their mechanical pushing effect [6]. Rowley et al. in a study on 179 cases reported the coexistence of more than one type of hamartoma in 30% of patients. The relatively flat hamartoma was most frequently observed (70%), followed by the mulberry-like lesion (55%) and the transitional type (9%). No correlation was found between the presence and type of hamartomas and age [72].

Other features of the retina found in TSC are retinal pigmentary changes including both hyperpigmented areas (possibly due to congenital retinal pigment epithelium hypertrophy) and areas of hypopigmentation with a “punched out” appearance that can be found both at the posterior pole and in the midperiphery [74]. Rowley et al. found a statistically significant correlation between punched out areas of chorioretinal hypopigmentation and TSC and showed 39% in the afflicted patients against 6% among controls [72].

Shields et al. studied retinal hamartomas with time domain OCT in 15 patients and found typical characteristics such as hyperreflectivity and “round, confluent, moth-eaten empty spaces with posterior shadowing” on the surface of the tumor and disorganization of the internal retinal layers. Moreover, the authors observed a gradual progression from normal to tumorous retina in all cases, inner retinal disorganization in 3 cases (20%), outer retina disorganization in 0 cases (0%), and disorganization of the entire retina in 5 patients (33%). Furthermore, OCT showed moderate retinal traction over the tumor in 4 cases, optically empty spaces in 10 cases, and shadowing posterior to the tumor in 14 cases (93%). Shallow elevation or edema of the retina adjacent to the tumor was found in 13% and 27% of cases, respectively, and macular edema was found in 20% of cases [12]. Soliman et al., using time domain OCT, differentiated OCT findings in the younger tuberous bodies where alterations were localized at the retinal nerve fiber layer with normal structure of the retinal layers and retinal pigment epithelium below, from OCT characteristics of older tuberous bodies where there was hyperreflectivity in the inner retinal layers with total shadowing of the underlying layers [75]. The translucent nature of the younger tuberous bodies could explain the absence of shadowing in these lesions [76]. Xu et al. showed that small retinal astrocytic hamartomas, undetectable with ophthalmoscopy, could be identified with infrared imaging and spectral domain OCT [77].

**Figure 11:**
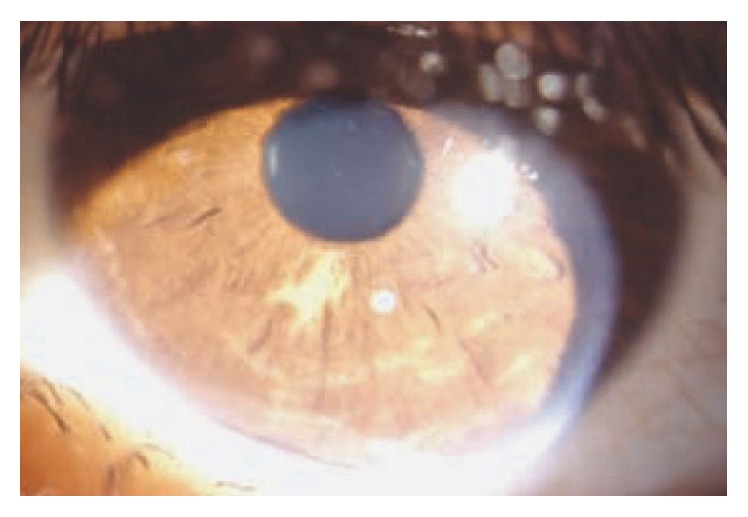
Slit lamp photograph of the anterior segment in a patient with tuberous sclerosis complex. A hypopigmented “ash leaf” shaped area is visible on the iris (from [6]).

Other ocular findings reported by Rowley et al. included palpebral angiofibromas in 39% of patients, nonparalytic strabismus in 5% (4 exotropia and 1 esotropia), iris and choroidal colobomas in 3%, and areas of iris depigmentation in 2%. Myopia was present in 27%, hyperopia in 22%, and astigmatism (>0.75 D) in 27% of patients; such distribution of refractive errors is fairly similar to the distribution among normal individuals, as assessed by population studies [72]. Lopez et al. reported a case of unilateral eyelid angiofibroma with complete blepharoptosis as the presenting sign of TSC [78]. Several case reports document the association between TSC and cataract [78, 79], serous retinal detachment [74, 80], hamartomas of the iris, and hamartomas of the ciliary epithelium [81]. In some cases “ash leaf” shaped iris hypopigmentation has been observed, as shown in a patient observed in our institute [6] (Figure 11). Ocular signs rarely described include corneal leukoma, megalocornea, primary and secondary glaucoma, optic nerve atrophy, papilledema, and VI nerve palsy [82].

Table 1 summarizes the clinical findings and diagnostic procedures in TSC.

In a review by Mennel et al., flat lesions evolved to more prominent forms without symptoms in about 10% of patients but spontaneous regression was also reported [83]. Therefore, observation is only necessary unless complications arise. In cases where exudation occurs, argon laser photocoagulation and photodynamic therapy have been used [83–85]. Saito et al. used intravitreal bevacizumab to treat two patients with tumor neovascularization and macular edema and found encouraging results as visual acuity and macular edema rapidly improved, tumor size decreased, and tumor associated neovascularization was rapidly attenuated [86].

Retinal toxicity has been associated with the drug vigabatrin, which is often used as first-choice therapy in the treatment of refractory seizures typical of TSC [87]. Retinal toxicity is shown with visual field loss that initiates as a bilateral nasal defect and evolves to concentric vision loss [88]. OCT of the peripapillary RNFL has shown that the alterations predominantly involve the nasal, superior, and inferior sectors [89]. Electroretinogram examination has been held as the optimal method to evaluate vigabatrin toxicity in children under nine years of age where perimetric examination is not reliable [87], but this frequently requires general anesthesia as some children cannot collaborate due to their young age or neurological condition. Thus, OCT has been proposed as a method to monitor therapy. In young children hand-held OCT, especially with the eye tracker system provided by spectral domain technology, is a rapid examination during general anesthesia [11, 90, 91]. Ophthalmic monitoring is advised upon initiation of vigabatrin therapy and every three and six months during treatment in children and adults, respectively; treatment should be suspended if there are signs of retinal toxicity [88].

## 4. Von Hippel-Lindau Disease

**Figure 12:**
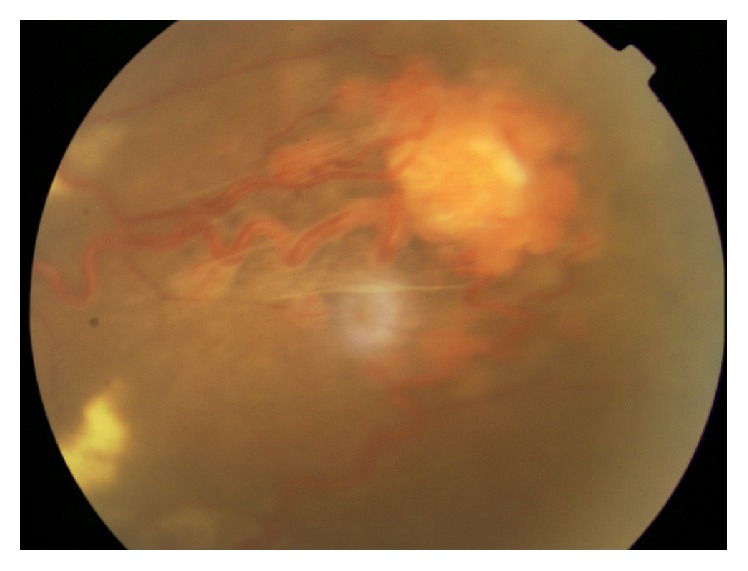
Fundus photograph of a retinal hemangioblastoma in a patient with Von Hippel-Lindau disease.

Von Hippel-Lindau (VHL) disease has an autosomal dominant transmission and is caused by a germline variation of the VHL tumor suppressor gene positioned on the short arm of chromosome 3p25-26 [92, 93]. Approximately 1/40,000 cases occur per year, and by age 65 years, there is close to 100% penetrance [92]. The condition is marked by the growth of numerous cysts and benign or malignant tumors in many organs. The most common characteristics of this disease are vascular tumors of the central nervous system hemangioblastoma (HB) and retinal capillary hemangioblastoma (RCH) also referred to as hemangioma. Lesions are found more commonly in the second or third decade of life although RCHs usually occur a decade earlier than cerebellar hemangioblastomas. Increase in vascular endothelial growth factors in VHL syndrome is believed to heighten RCH formation and growth [94, 95]. The most common and, most often, earliest manifestations of VHL disease are RCH, which occur in 43 to 85% of patients [96] (Figure 12).

RCH are either benign vascular neoplastic processes or congenital lesions [97]. They develop from the age of 10 until 30 years similarly to HB in the central nervous system. The frequency of RCH decreases progressively after the age of 30 years. RCH may remain asymptomatic for years and can regress spontaneously, but usually their growth leads to visual deterioration. They may be single or multiple tumors and are sometimes the only manifestation of the disease. Generally only a single tumor develops in one eye and it is usually asymptomatic in most patients [96]. Histopathological procedures show that RCHs are made of capillary blood-filled spaces lined by pericytes and endothelial and stromal cells [98]. These cells are similar to those found in renal cell carcinoma and cerebellar hemangioblastoma. Based on immunohistochemical studies, it has been advanced that this type of cell may represent lipidized fibrous astrocytes and glial cells [99] or more likely vasoformative stem cells [100] that may originate from angioblasts arrested in development [101].

On ophthalmoscopic examination, RCHs are confined, globular, red-orange vascular tumors, typically observed in the juxtapapillary or the peripheral retina. They vary in size from 10 to 3000 microns or more. Peripheral lesions are most commonly found in the superior-temporal quadrant [102]. In 15% of cases RCHs are in or around an area measuring one disc diameter from the optic disk. The lesions usually have a feeder vessel where exudation and subretinal fluid can be found. Ocular complications include macular or extramacular exudation, tractional retinal detachment, new vessels on a peripheral hemangioblastoma, and neovascular glaucoma [103].

**Figure 13:**
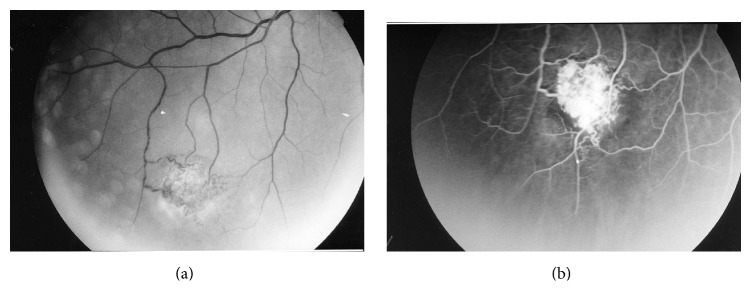
Fluorescein angiography images of a peripheral retinal hemangioblastoma in a patient with Von Hippel-Lindau disease. (a) Early red free image. (b) Late image (from [6]).

Small peripheral RCHs are sometimes difficult to detect with funduscopy and are better seen with fluorescein angiography where lesions are characterized by hyperfluorescence. The feeder vessel will hyperfluoresce and is well evidenced in the arterial phase whereas the draining vein is clearly seen in the venous phase. In the late phases the tumor shows hyperfluorescence and leakage (Figure 13).

Vessels in HB are fenestrated leading to exudation/leakage in contrast to normal retinal vessels. The differential diagnosis of RCH is with Wyburn-Mason disease (racemose hemangioma), Coats' disease, retinal macroaneurysm, vasoproliferative retinal tumor, and retinal cavernous hemangioma [102].

Shields et al. described OCT characteristics of RCH, which showed thickening and structural alterations of the retina with mild shadowing of the underlying structures. These authors highlighted the importance of OCT in evaluating intra- and subretinal fluid; longstanding retinal edema in these lesions had features similar to cystoid macular edema whereas the chronic persistence of subretinal edema seemed to have caused a reduction of thickness of the layer corresponding to photoreceptors [104]. Rarely exophytic RCHs arising from the outer retina, which do not commonly have arteriovenous shunting, have been reported in VHL disease. OCT features observed in these forms are location of the lesions in the outer retinal layers, shadowing, and photoreceptor layer rips close to the lesion [105]. OCT is also useful in evaluating the evolution of small lesions. The setback for OCT is when lesions are large and cannot be scanned due to technical limitations of the instrument [106].

Table 1 summarizes the clinical findings and diagnostic procedures in VHL disease.

Treatment of RCH usually includes laser photocoagulation, photodynamic therapy, cryotherapy, radiation, and surgical removal. In the literature various authors recommend early treatment of RCH when lesions tend to enlarge and become more difficult to treat with time [107]. Nevertheless, asymptomatic peripheral RCH lesions only require observation and treatment only in case of complications.

Singh et al. describe their experience in the management of RCH in 77 eyes of patients with VHL. Eighty-two percent of RCH were initially observed for a period of 84 months and the authors suggested only close observation in lesions smaller than 1500 microns, which are not visually threatening [107, 108]. Photocoagulation with argon, krypton, and yellow dye and diode laser is effective in extrapapillary or juxtapapillary RCH where lesions are 1500 microns or less in size and localized in the posterior retina [107, 108]. Cryotherapy has been used for the treatment of extrapapillary RCHs [105]. Singh et al. performed cryotherapy in lesions larger than 3000 microns and treated extrapapillary RCH with a mean size of 4500 microns with iodine-125 plaque with good results [108]. Thermotherapy, external bean radiotherapy, pars plana vitrectomy, and related procedures are more frequently used to treat the juxtapapillary locations of RCH [108].

Photodynamic therapy (PDT) is aimed at vascular endothelial cells and has also been employed in the management of RCH [109, 110]. However, when a hemangioblastoma is associated with the optic nerve, such treatment can result in loss of vision. Therefore, VEGF-targeted strategies have been tested but therapy outcomes are contrasting [107, 111, 112]. SU5416 is an inhibitor of VEGF receptor-2, which was used through intravenous administration in a case of RCH of the optic nerve head and in a single case of a juxtapapillary RCH and improved visual acuity and visual field without reducing the size of the tumor [113, 114]. Madhusudan et al. found stability or improvement in the ocular lesions in 2 out of 6 patients treated with SU5416 [115]. Intravitreal bevacizumab associated with photodynamic therapy showed tumor regression and reduction of exudation [112], whereas pegaptanib only reduced exudation with no effect on tumor size [116]. Wong et al. published their results on 5 cases treated with intravitreal ranibizumab and reported that in two patients with large juxtapapillary lesions associated with circinate lipid, intraretinal hemorrhage, and marked retinal edema extending into the macula this therapy did not exert any beneficial effect whereas it was successful in a patient with a juxtapapillary endophytic lesion of one disk diameter with limited retinal edema not involving the fovea [111]. They concluded that intravitreal ranibizumab does not have a beneficial action on the largest and most exudative lesions but is efficacious in small lesions [111]. Therefore, effectiveness and appropriateness of treatment are influenced by the site and size of RCH; treatment cannot be delivered to juxtapapillary RCHs without damage to the optic nerve whereas it can be applied to the peripheral retina [117]. Today intravitreal anti-VEGF treatment is successfully used in a wider array of retinal pathologies and recently in metastatic tumors of the choroid [118–122]. A principal motive for unsuccessful outcome of this treatment may be the size of hamartomatous lesions in VHL disease, which can be considerably greater than choroidal neovascular membranes [123]. Moreover, the mature vasculature of the hamartomas and genetic alterations underlying the phakomatoses leading to incessant production of VEGF can result in poor response [111, 123].

## 5. Conclusions

The phakomatoses require a multidisciplinary approach and the ophthalmologist plays a fundamental role in diagnosis and management. Imaging techniques in ophthalmology have made groundbreaking progress in the past decade and NIR may lead to the inclusion of choroidal nodules as the 8th diagnostic criteria in NF1. OCT provides novel information on retinal hamartomas and enhanced depth imaging technology enables visualization of the choroid in vivo providing further information on the morphology of the choroid and choroidal nodules in NF1. Peripapillary RNFL analysis with OCT is a noninvasive tool and is an adjunct in monitoring optic pathway gliomas. Furthermore, hand-held OCT enables faster examination of the RNFL in children who are unable to collaborate due to their young age and neurological conditions. An enormous bulk of research has led to the establishment of optimal doses and protocols for intravitreal anti-VEGF treatment in retinal pathology, but little information is available for this treatment in the phakomatoses and reports on the use of anti-VEGF agents are contrasting and incomplete. The future might hold new drug delivery systems for the intravitreal treatment of symptomatic retinal hamartomas.

## Figures and Tables

**Table 1:** Clinical findings and diagnostic procedures in the phakomatoses.

	Major ophthalmic features		Minor ophthalmic features	
	Clinical findings	Diagnostic procedures	Clinical findings	Diagnostic procedures
NF1	Lisch nodules	*Slit lamp examination* *Anterior segment OCT * *UBM*	Pulsating proptosis	*Slit lamp examination* *Angio-MRI*
	Optic pathway gliomas	*MRI* *Visual field examination* *Optic nerve head evaluation* *RNFL OCT*	Microphthalmus/enophthalmos	*Slit lamp examination* *Ultrasound scans* *MRI*
	Choroidal alterations	*NIR/OCT* *ICGA*	Upper eyelid plexiform fibroma	*Slit lamp examination* *Excisional biopsy/histopathology*
			Conjunctival neurofibroma	*Slit lamp examination* *Excisional biopsy/histopathology*
			Hypertrophic corneal nerves	*Slit lamp examination* *Confocal microscopy*
			Glaucoma	*Slit lamp examination* *Tonometry* *Gonioscopy* *Visual field examination* *Optic nerve head evaluation* *UBM* *RNFL OCT*
			Microvascular retinal abnormalities	*Fundus examination* *NIR* *FAG*

TSC	Retinal astrocytic hamartomas (elevated, transitional, smooth)	*Fundus examination* *FAG* *OCT*	Retinal pigmentary changes	*Fundus examination*
			Palpebral angiofibroma	*Slit lamp examination* *Excisional biopsy/histopathology*
			Iris/choroidal coloboma	*Slit lamp examination* *UBM* *Fundus examination*
			Iris depigmentation	*Slit lamp examination*

VHL	Retinal capillary hemangioblastoma	*Fundus examination* *FAG* *OCT (secondary macular edema)*	—	—

FA: fluorescein angiography; ICGA: indocyanine green angiography; MRI: magnetic resonance imaging; NF1: neurofibromatosis type 1; NIR: near infrared imaging; OCT: optical coherence tomography; RNFL OCT: retinal nerve fiber layer optical coherence tomography; TSC: tuberous sclerosis complex; UBM: ultrasound biomicroscopy; VHL: Von Hippel-Lindau disease.
